# Supplementary material for: The Xenopyricularia zizaniicola exhibits a genome architecture distinct to the two-speed genome
Source: Microbiol Spectr. 2025 Jun 9;13(7):e00362-25. doi: 10.1128/spectrum.00362-25 (PMC12210920; doi:10.1128/spectrum.00362-25)
Supplement: Figures S1 and S2 — Fig. S1: The intergenic length. Fig. S2: Distance from genes to repeats. [file spectrum.00362-25-s0001.pdf]

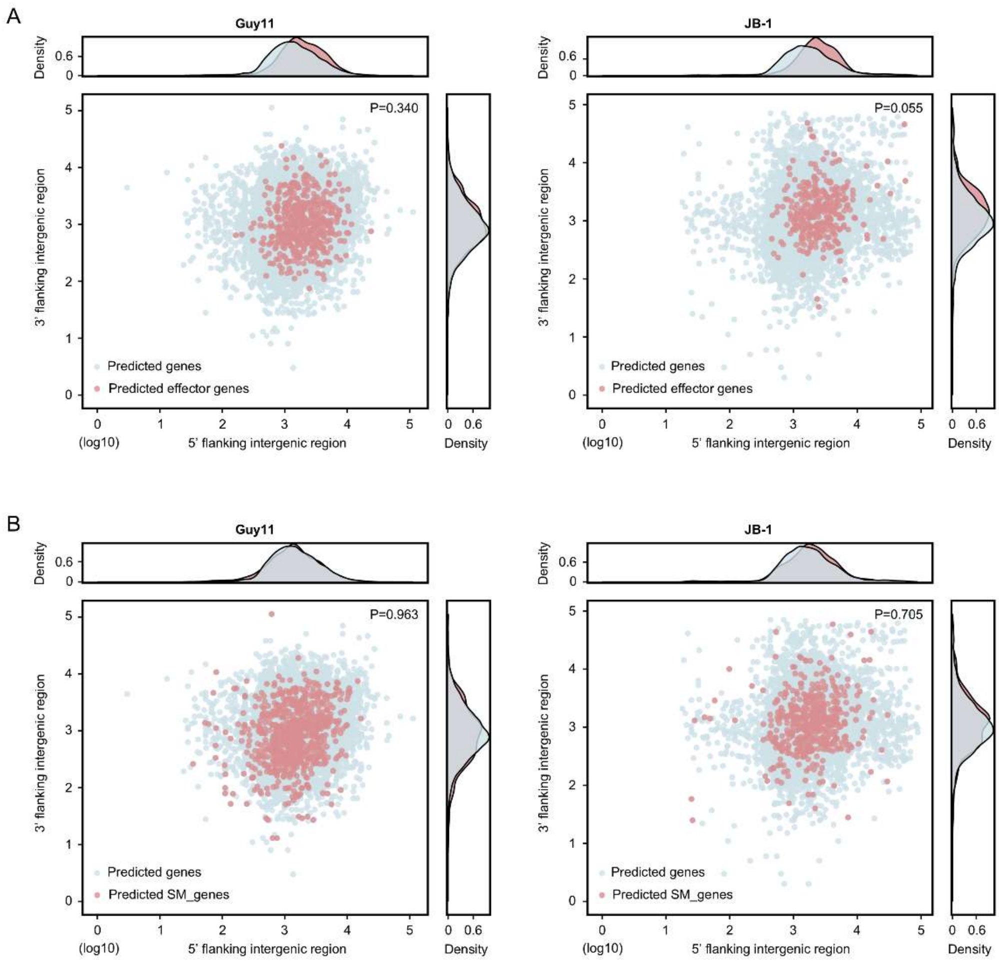

FIG S1 The intergenic length of *P. oryzae* strain Guy11 and *X. zizaniicola* strain JB-1. (**A**) The intergenic length of predicted effector genes and other genes. (**B**) The intergenic length of predicted secondary metabolite biosynthesis genes (SM_genes) and other genes. Statistical analysis: 100 each group of genes were randomly sampled 1,000 times for both the 5′ and 3′ intergenic length. Significance across 2,000 subsets was assessed via Mann–Whitney *U* tests with Benjamini–Hochberg correction. The final *P*-value represents the mean of corrected *P*-values from 5' and 3' intergenic length.

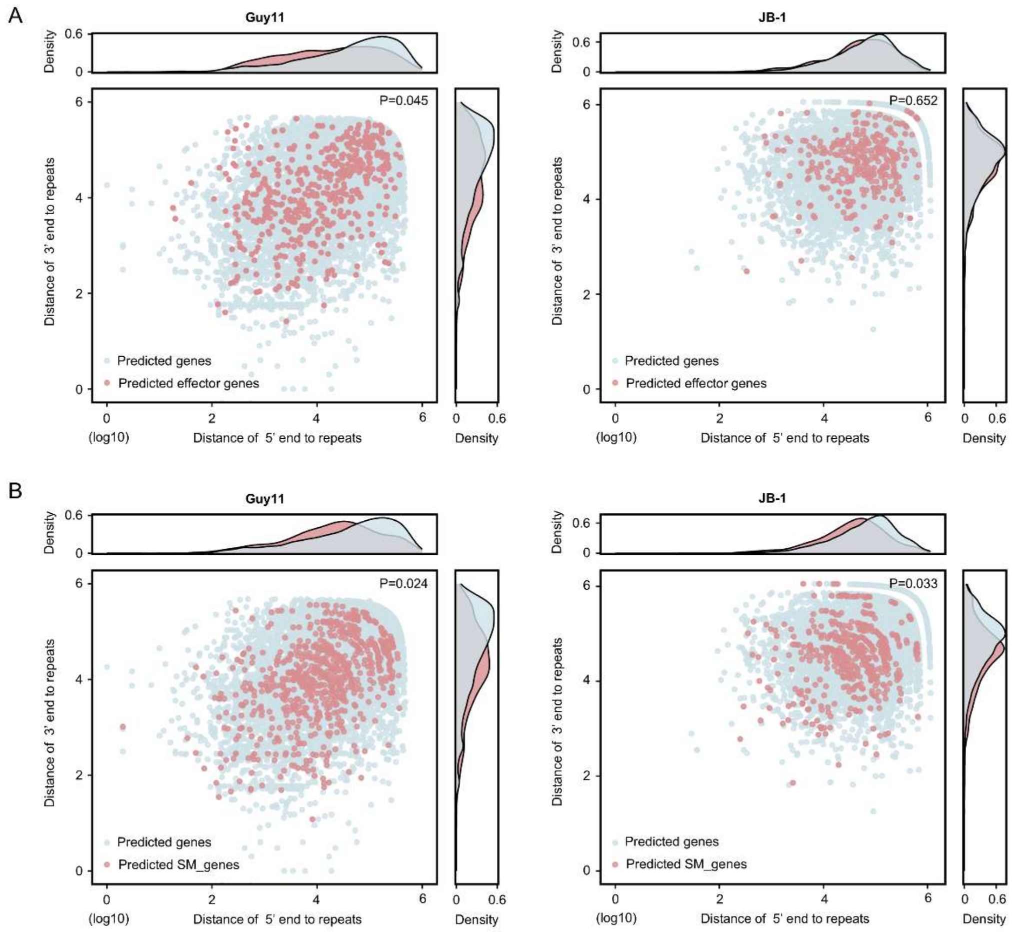

FIG S2 Distance from the 5′ and 3’ ends of predicted effector genes and secondary metabolite biosynthesis genes (SM_genes) to repeats in *P. oryzae* strain Guy11 and *X. zizaniicola* strain JB-1. (**A**) The 5′ and 3′ ends distance of predicted effector genes to repeats. (**B**) The 5′ and 3′ ends distance of predicted SM_genes to repeats. Statistical analysis: 100 each group of genes were randomly sampled 1,000 times for both the 5′ and 3′ distances to repeats. Significance across 2,000 subsets was assessed via Mann–Whitney *U* tests with Benjamini–Hochberg correction. The final *P*-value represents the mean of corrected *P*-values from 5' and 3' distances to repeats.
